# The Effect of Ambient Temperature on *Brachypodium distachyon* Development

**DOI:** 10.3389/fpls.2019.01011

**Published:** 2019-08-21

**Authors:** Meixia Li, Alice Kennedy, Michiel Huybrechts, Niklas Dochy, Koen Geuten

**Affiliations:** Department of Biology, KU Leuven, Leuven, Belgium

**Keywords:** ambient temperature, heading time, seed maturation time, seed weight, seed dormancy

## Abstract

Due to climate change, the effect of temperature on crops has become a global concern. It has been reported that minor changes in temperature can cause large decreases in crop yield. While not a crop, the model *Brachypodium distachyon* can help to efficiently investigate ambient temperature responses of temperate grasses, which include wheat and barley. Here, we use different accessions to explore the effect of ambient temperature on Brachypodium phenology. We recorded leaf initiation, heading time, leaf and branch number at heading, seed set time, seed weight, seed size, seed dormancy, and seed germination at different temperatures. We found that warmer temperatures promote leaf initiation so that leaf number at heading is positively correlated to temperature. Heading time is not correlated to temperature but accessions show an optimal temperature at which heading is earliest. Cool temperatures prolong seed maturation which increases seed weight. The progeny seeds of plants grown at these cool ambient temperatures show stronger dormancy, while imbibition of seeds at low temperature improves germination. Among all developmental stages, it is the duration of seed maturation that is most sensitive to temperature. The results we found reveal that temperature responses in Brachypodium are highly conserved with temperate cereals, which makes Brachypodium a good model to explore temperature responsive pathways in temperate grasses.

## Introduction

As sessile organisms, plants have evolved strategies to cope with environmental conditions, including changes in temperature. Plants have a characteristic optimal growth temperature, above or below which, growth and development can be strongly affected but the plant can still survive (Schlenker and Roberts, 2009; Zhao et al., 2017; Tigchelaar et al., 2018). These non-stressful temperatures have been defined as the ambient temperature range (Capovilla et al., 2015). Even though these temperatures are technically non-stressful, in the case of crops, the yield can still be significantly affected by such temperature changes. Models have projected that the yield of wheat, maize, soybean and rice can decrease by 6, 7.4, 3.1, and 3.2%, respectively, with only a 1°C increase in the global temperature (Zhao et al., 2017). While a one-degree change in mean temperature might be realized through changes in the temperature extremes, also minor changes in temperature can affect the growth and development of plants and crops (Porter and Gawith, 1999). The effect of the ambient temperature on plants has become a global focus of research in not only the model plants but also the crops. To improve crop resilience to a changing climate, the molecular mechanisms underlying plant developmental responses to ambient temperature need to be understood.

The effect of ambient temperature on all developmental stages of the model system *Arabidopsis thaliana* (henceforth Arabidopsis) is now well understood. Growth and developmental effects of high ambient temperatures during seedling development are summarized in a review by Quint et al. (2016). Briefly, the elongation of the hypocotyl, petiole and root, and hyponastic growth increase in elevated temperatures, whereas stomatal density and leaf thickness decrease at high ambient temperatures. Interestingly, hyponasty, together with petiole elongation results in an open rosette structure to cool down the growth environment at high ambient temperatures (Crawford et al., 2012). For the flowering response to ambient temperatures, it is concluded generally that elevated temperatures accelerate flowering time, despite natural variation (Thingnaese et al., 2003; Lempe et al., 2005; Balasubramanian et al., 2006; Ibañez et al., 2017). As well as that, plants grown at low ambient temperatures produce more leaves by flowering time than those grown at high ambient temperatures, because they flower later and have more time to produce leaves (Blázquez et al., 2003; Samach and Wigge, 2005; Lee et al., 2007). For seed development, high ambient temperatures shorten the time to set seeds and decrease seed yield in Arabidopsis (Springthorpe and Penfield, 2015). Moreover, a high ambient temperature during seed development decreases the seed dormancy compared to low ambient temperatures through the regulation of seed coat phenylpropanoid metabolism (MacGregor et al., 2015), while high ambient temperatures during seed imbibition prior to germination decrease the rate of seed germination (Huang et al., 2014; Springthorpe and Penfield, 2015).

The genetic regulation of the ambient temperature responses at each developmental stage in Arabidopsis have also been studied in great detail. At the seedling stage, the photoreceptor PHYTOCHROME B (PHYB) was reported to regulate the temperature-dependent hypocotyl growth under short days (Halliday et al., 2003; Legris et al., 2016). The temperature-sensing activity of PHYB is mediated through the transcriptional activator HEMERA (Qiu et al., 2019). In addition, temperature-responsive flowering in Arabidopsis has been shown to be regulated by *SHORT VEGETATIVE PHASE* (*SVP*) and *FLOWERING LOCUS C* (*FLC)* clade genes including *FLC*, *FLOWERING LOCUS M* (*FLM*), and *MADS AFFECTING FLOWERING* (*MAF*) (Blázquez et al., 2003; Balasubramanian et al., 2006; Chiang et al., 2009; Gu et al., 2013; Posé et al., 2013; Lutz et al., 2015). The molecular regulation is mediated by the ambient temperature-dependent degradation of *SVP* and the alternative splicing of *FLM* and *MAF2*. *PHYTOCHROME INTERACTING FACTOR4* (*PIF4*) is also implicated in this response as an activator of the flowering promoter *FLOWERING LOCUS T* (*FT*) at high ambient temperatures (Kumar et al., 2012). *FLC* mRNA is suppressed by FT through activating antisense *FLC* transcripts during seed development (Chen and Penfield, 2018). Furthermore, *DELAY OF GERMINATION1* (*DOG1*) was reported to be important in temperature-dependent regulation of seed dormancy in order to adapt timing of germination in changing ambient temperatures. At low ambient temperatures, regulation of *DOG1* is mediated by C-REPEAT BINDING FACTOR (CBF) (Kendall et al., 2011). Despite the wealth of knowledge on ambient temperature-dependent regulation of development in Arabidopsis, there are many fundamental developmental and geographical differences to consider when applying this to temperate cereals. Cereal-specific developmental responses to ambient temperatures need to be considered.

The ambient temperature-response of cereals has already been well documented. The temperature effect on wheat was summarized in an early review (Porter and Gawith, 1999). Below a certain threshold, leaf initiation and emergence, leaf number and shoot elongation are promoted by high ambient temperatures, however, no linear temperature response from emergence to anthesis was found (Angus et al., 1981). Yet, temperate cereals show highly conserved temperature responses in seed traits. Specifically, high ambient temperatures result in short seed set time, low seed yield, weak dormancy and early germination (Al–Khatib and Paulsen, 1984; Wardlaw and Moncur, 1995; Ferris et al., 1998; Nasehzadeh and Ellis, 2017). Despite numerous research studies on phenotypic responses of cereals to temperature, the molecular regulation of these responses is significantly less well-known when compared to the information available for Arabidopsis. In barley, *MYELOBLASTOSIS* (*MYB*) circadian clock related genes such as *CIRCADIAN CLOCK ASSOCIATED 1* (*CCA1*), *GIGANTEA* (*GI*), *LUX ARRHYTHMO* (*LUX*) are increased at high ambient temperatures (Ford et al., 2016) and photoperiod response genes *PHOTOPERIOD 1* (*Ppd-H1*) and *EARLY FLOWERING 3 (ELF3)* are involved in the temperature-dependent reproductive development (Ejaz and von Korff, 2017). *HvODDSOC2* and *VER2*-like genes are possible candidates for high-temperature-responsive regulators in barley (Hemming et al., 2012). As well as that, an *earliness per se* QTL on *Triticum monococcum* chromosome 1Am was reported to show a significant interaction with temperatures on flowering time (Bullrich et al., 2002). *MOTHER OF FT AND TFL1* (*MFT*) was reported to be upregulated in dormant seeds produced at lower temperatures (Nakamura et al., 2011), while *MFT* in Arabidopsis promotes embryo growth through a feedback loop in ABA signal pathway (Xi et al., 2010). Moreover, the floral repressor *VERNALIZATION2* (*VRN2*), and *ODDSOC2*, two *MADS*-box genes in wheat, were reported to be re-activated in high ambient temperatures after vernalization, but the regulatory mechanism is unclear (Yan et al., 2004; Kiss et al., 2017; Dixon et al., 2019).

Despite similarities and advances in the genetic understanding of ambient temperature effects on plant growth, Arabidopsis is still limited as a model for the temperate grasses. Particularly because temperature responses are adaptive traits which can evolve quickly, it can be expected that the molecular control of these developmental responses is significantly different. The reason why the molecular mechanisms of ambient temperature responses of temperate cereals remain elusive may be owed to the fact that cereals generally have larger more complex genomes, they are a lot more difficult to work with in a practical sense and there is usually reduced availability of mutant material. Therefore, a more suitable model for temperate cereals is required to increase the rate at which information on the molecular regulation of ambient temperature-dependent responses in cereals can be obtained. *Brachypodium distachyon* (2*n* = 10), a diploid temperate grass, has been chosen as a model for temperate grasses because of its small, diploid genome (∼355 Mb), practical growth size and efficient transformation (Brkljacic et al., 2011). The ambient temperature effects on the development and growth have been studied in Brachypodium. 9% of the transcriptome in accession Bd21 was significantly different when shifted from cool temperature (12°C) to warm (22°C) and to high (27°C) temperatures (Boden et al., 2013). A high percentage of heat shock genes were identified at high temperature (27°C), which may be a stressful temperature for Bd21 (Boden et al., 2013). During seed development, the stability of H2A.Z, one of the histone variants, determines the seed yield in Brachypodium (Boden et al., 2013). Also, *BdVIL4*, a *VERNALIZATION INSENSITIVE 3* like gene in Brachypodium, was proven to inhibit flowering and branching responding to ambient temperatures through repressing miRNA156 (An et al., 2015). Despite this, the basic developmental responses to ambient temperatures in Brachypodium have not been extensively described. Here, we use different Brachypodium accessions to explore the phenotypic plasticity of ambient temperature responses from leaf development to seed germination.

## Materials and Methods

### Plant Material and Growth Condition

We selected five natural accessions of *B. distachyon*, Bd21, Bd21-3, Mon3, Bd1-1, and BdTR3C. The accessions flowering faster with vernalization were previously indicated as facultative accessions, including Bd21, Bd21-3, and Mon3, while the accessions that could not flower without vernalization were indicated as winter accessions, including BdTR3C and Bd1-1 (Colton-Gagnon et al., 2014; Ream et al., 2014). The geographical origin (Des Marais et al., 2017; Stritt et al., 2017) and the local climate data of these five accessions including the average precipitation and the average temperature for every month was collected using New_LocClim software of FAO (Grieser et al., 2006).

For the vegetative stage experiment, seeds were vernalized at 4°C for 2 weeks (Bd21, Bd21-3, Mon3) or 6 weeks (BdTR3C) in soil before being moved to a Lovibond growth chamber at either 18°C or 26°C, a day/night light cycle of 16/8 h and an LED light intensity of 100 μmol m^–2^ s^–1^. Thirty plants from each accession were used for phenotyping in each temperature condition.

For the reproductive development experiment, plants were grown at 22°C for 3 weeks in a Lovibond growth chamber with a 16/8 h day/night cycle and a light intensity of 60 μmol m^–2^ s^–1^ before being vernalized at 4°C for 2 weeks (Bd21, Bd21-3, and Mon3) or 6 weeks (BdTR3C and Bd1-1) with an 8/16 h day/night cycle and a light intensity of 20 μmol m^–2^ s^–1^. After vernalization, plants were moved to a Lovibond growth chamber at either 14°C, 18°C or 22°C, 16/8 h, LED light 60 μmol m^–2^ s^–1^. Fifteen plants from each accession were used in each temperature condition.

For the seed dormancy experiment, physiologically mature seeds from the reproductive development experiment were incubated at 12°C. At least five replicates with twenty seeds from each accession were used in this experiment.

For the seed germination experiment, plants were grown at 25°C, 16/8 h for 3 weeks before being vernalized at 4°C, 8/16 h for a number of weeks to saturate their vernalization requirement and then moved to a growth chamber at 20°C 16/8 h until seed maturation. Physiologically mature seeds or after-ripened seeds from twenty natural accessions were incubated at 12°C or 18°C. Twenty seeds from each accession were used in each temperature condition.

### Phenotype

For leaf development during the vegetative stage, total leaf numbers of the whole plant were counted two times per week after 2-week growth. Leaf number divided by the days were defined as leaf development rate per day in this research.

For the reproductive experiment, heading time, total leaf number when heading, branch number when heading, seed harvest time (physiological maturity), dry seed weight (50 seeds) and seed area for each plant were recorded. The seed set time is defined from heading to seed physiological maturity. To measure seed weight, 150 seeds from each temperature condition were dried at 37°C for 4 weeks (50 de-husked seeds for one replicate). GrainScan was used to measure the seed area (same samples for seed weight) based on the protocol of GrainScan (Whan et al., 2014).

Multiple comparison of one-way ANOVA was used for the statistical analysis for phenotypic data at different temperatures within one accession.

### Seed Germination

According to the BBCH scale for Brachypodium (Onda et al., 2015), physiologically mature seeds (when the spikelets lose all greenness, BBCH93) were collected in tubes and stored at −80°C. This freezing method arrests all metabolic processes, ensuring the spikelets kept their current dormancy levels.

For the seed dormancy experiment, physiologically mature seeds from the reproductive development experiment were used. Twenty de-husked seeds from each accession were put on 0.5% agar plates after sterilization via fumigation [100 ml bleach: 3 ml (37%) HCL]. Twenty seeds from each plant (considered as one replicate) were incubated on agar plates at 12°C in darkness after sterilization. At least five replicates (Bd21 14°C *n* = 5, Bd21 18 and 22°C *n* = 10, Bd21-3 18 and 22°C *n* = 10, Mon3 14, 18 and 22°C *n* = 10, BdTR3C and Bd1-1 *n* = 10) from each temperature condition were used in this research. When the first sign of radicle protrusion was observed, the number of germinated seeds was noted. Seed germination was checked every 24 h with green light and the number of germinated seeds was recorded for 10 days. One-way ANOVA was used for the statistical analysis for phenotypic data at different temperatures within one accession.

For the seed germination assay, the degree of maturity of the spikelets was checked twice a week. Seeds were harvested at three time points according to their level of maturity: physiologically mature, 2- (PM + 2) or 4-weeks (PM + 4) post-physiological maturity. At least 10 de-husked seeds from each plant were incubated on agar plates at 12°C or 24°C in darkness after sterilization as described previously. The number of germinated seeds per day was recorded. At least 15 accessions were used to calculate the germination rate index (GRI). The accessions and the seed numbers used for the seed germination experiment were summarized in Supplementary Table S1.

To quantify the seed dormancy, the germination percentage (G) and GRI were calculated (Al-Mudaris, 1998). G presents the dormancy ability and was calculated by the total germinated seeds divided by total seeds used for dormancy experiment. GRI presents the germination speed and was calculated by the following formula:

G⁢R⁢I=G⁢1/1+G⁢2/2+…+G⁢i/i

where *Gi* = germination percentage on day *i*.

In order to know the germination time, the mean germination time (MGT) (Supplementary Figure S1) was calculated by the following formula (Al-Mudaris, 1998):

M⁢G⁢T=Σ⁢(F×X)/Σ⁢F

where Σ(*F*×*X*) = number of seeds germinated on day *X*.

### Q10 Temperature Coefficient

To quantify the temperature effect among different accessions for a certain trait, the Q10 temperature coefficient was calculated. The Q10 temperature coefficient is widely used to represent the temperature sensitivity of traits among different accessions, which represents a trait change if ambient temperature increases by 10°C (Hegarty, 1973). Thus, Q10 can test the genotype effects by temperature of a certain trait. High variation of Q10 values for one trait means the temperature responses are less conserved among accessions for this trait. Q10 value was calculated to represent the natural variation for certain traits during reproductive stage. The Log (Q10) value were used to visualize the data. The Q10 temperature coefficient is calculated by the following formula:

$$\text{Q}\,10={\left(\frac{P\,w}{P\,c}\right)}^{\frac{10}{T\,w-T\,c}}$$

where *Pw*, trait value at warm temperature; *Pc*, trait value at cold temperature; *Tw*, warm temperature; and *Tc*, cold temperature.

## Results

### The Local Climate Limits the Growth Season of Brachypodium Accessions

**FIGURE 1:**
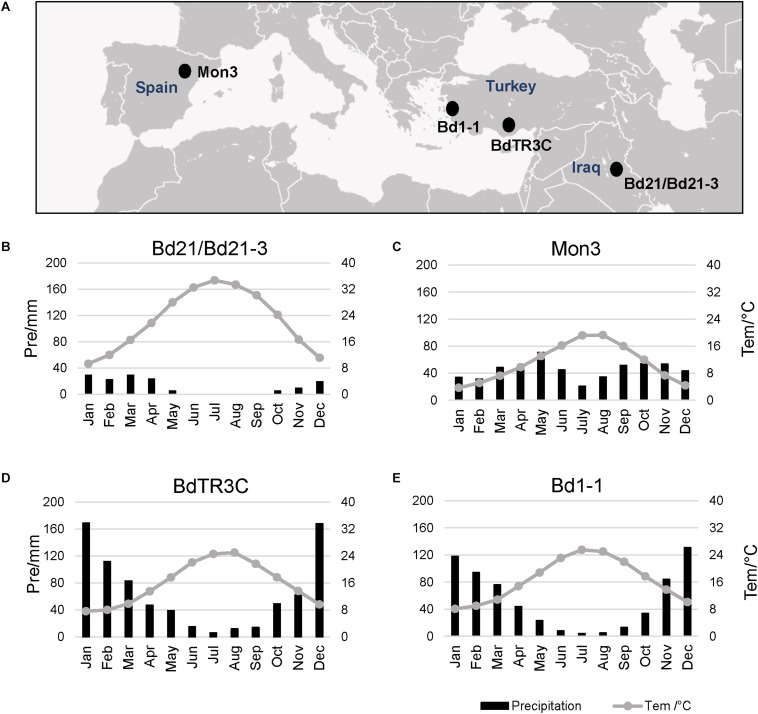
Local climate information for natural accessions of Brachypodium. Data from New LocClim software (Grieser et al., 2006). **(A)** Locations of different accessions. **(B–E)** Average precipitation (bar chart) and average temperature (line) of the whole year for each accession.

Five natural Brachypodium accessions distributed in the southern Eurasian area were used in this research, including facultative and winter accessions (Figure 1A). The natural climate for these accessions is mainly the classic Mediterranean type with a hot summer and wet winter, which limits the growth period for plants (Wilson et al., 2015; Des Marais et al., 2017). Perhaps due to the limited precipitation and hot summer, Bd21 and Bd21-3 appear to have a short life cycle with low dormancy and weak vernalization responses (Figure 1B). Mon3 grows in a climate where heat or precipitation do not seem to limit the growth season (Figure 1C). In spite of the hot summer, the adequate rainfall during winter can guarantee the growth of the winter accessions BdTR3C and Bd1-1 in spring (Figures 1D,E). Based on these data, we designed our experiments between 12 and 26°C to investigate the effect of ambient temperature on the different developmental stages of Brachypodium.

### Warmth Accelerates Leaf Initiation During the Early Vegetative Stage

**FIGURE 2:**
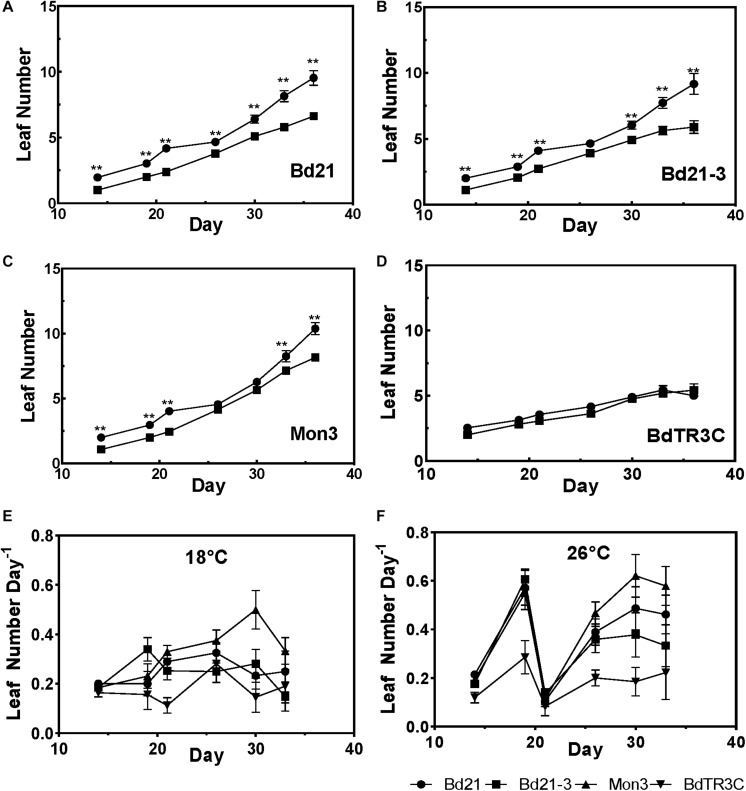
Leaf development in the vegetative stage at different temperatures. **(A–D)** Leaf numbers at 26°C (black circle) and 18°C (black square) for different accessions. **(E,F)** The leaf initiation rate at 18 and 26°C in different accessions. Student test was used for statistically analysis and ^∗∗^adjusted *P* < 0.01.

In order to investigate the effect of ambient temperature on Brachypodium vegetative development, four accessions (Bd21, Bd21-3, Mon3, and BdTR3C) were grown at both 18 and 26°C after being vernalized as seeds. In one comparison, we plotted the appearance of leaves over time (Figures 2A–D). We observed that a higher temperature promotes leaf development in Bd21, Bd21-3, and Mon3, while the winter accession BdTR3C shows no difference in leaf number. Possibly, and consistent with its winter habit, BdTR3C may not be sensitive to this range of temperatures during the vegetative stage and it is only responsive to colder temperatures (vernalization) in this developmental stage.

To investigate changes in the rate of leaf initiation, we plotted leaf number per day over time. Indeed, a higher temperature results in an increased leaf initiation rate, except for BdTR3C (Figures 2E,F). At 26°C, a synchronous growth pattern can be observed for the facultative accessions, in which leaf initiation is initially accelerated, suddenly decreased and subsequently restored. A similar pattern was not observed at 18°C. It is possible that facultative accessions are more sensitive to high ambient temperatures at the seedling stage. This needs to be investigated further with a larger panel of accessions because to our knowledge, a similar observation has not been made for wheat (Baker et al., 1986).

### Heading Is Accelerated at an Optimal Temperature

**FIGURE 3:**
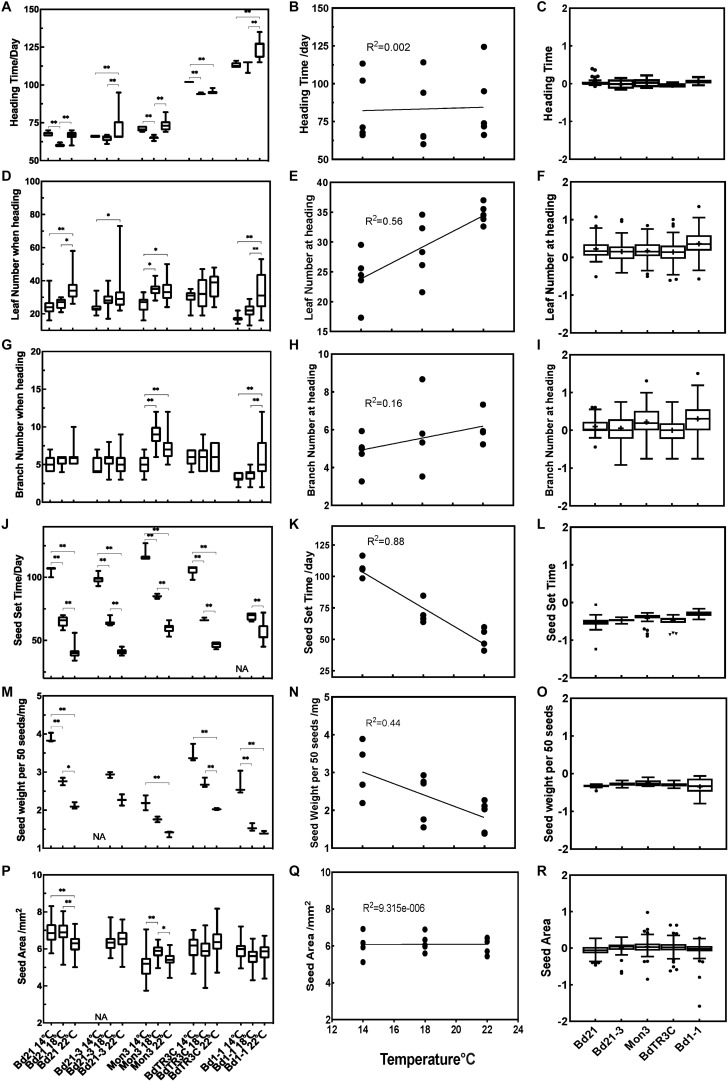
Trait effects during the reproductive stage at 14, 18, and 22°C. Left panels **(A,D,G,J,M,P)** phenotype data for different accessions for heading time, leaf number at heading, branch number at heading, seed set time, seed weight per 50 seeds and seed area. Middle panels **(B,E,H,K,N,Q)** correlation between traits and temperatures. Right panels **(C,F,I,L,O,R)** log (Q10) for different traits in different accessions. One-way ANOVA was used for statistically analysis and ^*^adjusted *P* < 0.05, ^∗∗^adjusted *P* < 0.01. NA, missing data.

To understand the effect of temperature on subsequent growth during the reproductive stage, we designed a second experiment. In this experiment, plants were grown at 22°C for three weeks before being vernalized for 2or 6 weeks as seedlings during which growth virtually stops. Plants were then moved to three different temperature conditions (14, 18, and 22°C) until seed maturity. Traits at heading (heading time, branch number and leaf number) and traits related to seeds (seed set time, seed weight per 50 seeds, seed area) were recorded. All the phenotypic values among different accessions are summarized in Figure 3, which includes the phenotypic data, the correlation with temperature and trait values, and the Q10 temperature coefficient, which quantifies the temperature sensitivity of a trait.

While heading time is affected by ambient temperature in every accession, this effect is not linear (Figure 3A). Rather, a response is observable in which an optimal temperature accelerates heading. This response is most pronounced for Bd21, and Mon3, in which 18°C accelerates heading in comparison to 14 and 22°C. Bd21-3 and Bd1-1 show only a minor difference between the lower temperatures, while a higher ambient temperature (22°C) delays heading. High temperatures (18 and 22°C) promote heading time in BdTR3C. There is no linear correlation between temperature and heading time among the five accessions (Figure 3B). The absence of a correlation between heading time and temperature can also be derived from the log(Q10) index, which is close to zero and shows small variance (Figure 3C). So while there are significant temperature effects for Brachypodium flowering time, these are relatively small in general and show an optimum rather than a linear correlation.

### Leaf Number at Heading Is Positively Correlated to Elevated Temperatures

As shown, higher temperatures accelerate leaf initiation and this is reflected in the number of leaves produced at the time of heading. Specifically, for Bd21, Mon3, and Bd1-1, leaf numbers at high ambient temperatures are significantly different from leaf numbers at the low ambient temperature, while Bd21-3 shows large variance in leaf numbers at high ambient temperature. No significant difference was observed in BdTR3C for different temperatures, which is similar to what we observed between 18 and 26°C (Figure 1D). This suggests that leaf development in BdTR3C is not sensitive to the temperature change. The correlation data shows that overall, leaf numbers are positively correlated to ambient temperature (*R*^2^ = 0.56) (Figure 3E). The log(Q10) plot confirms that there is a rather large variation in the temperature response of plants within accessions but that the response is positive overall (Figure 3F).

Branch numbers at heading are not as clearly affected by ambient temperature as leaf numbers. We use the term branch rather than tiller, because in most accessions in Brachypodium the branching does not start at the base but rather occurs along the stem (Jensen and Wilkerson, 2017). Branch numbers at heading did not show a statistical difference between temperatures in Bd21 and BdTR3C (Figure 3G), but there is a statistically significant difference in Mon3 and Bd1-1 between low ambient temperature and high ambient temperature (Figure 3G). These accessions also have a more bushy habit and generate more branches. Interestingly, there is a slight correlation trend between branch numbers and temperatures (*R*^2^ = 0.16) among all accessions (Figure 3H). Similar to what we observed for the log(Q10) plot of leaf numbers, branch numbers show large variation between plants, sensitivity was only observed for accessions Bd1-1 and Mon3 (Figure 3I), which generate more branches at higher temperature.

### Seed Maturation Time and Seed Weight Are Negatively Correlated to Temperature

Among all accessions, seed maturation time (also seed set time) and seed weight are strongly negatively correlated to temperature with low temperatures significantly prolonging the time taken for seed maturation and increasing seed weight (Figures 3J,M). Notably, 14°C is too low for Bd21-3 to fertilize normally and few seeds were produced as a result. Both seed maturation time and seed weight are negatively correlated (*R*^2^ = 0.88 and 0.44) to elevated temperatures (Figures 3K,N). This is also indicated by the negative log(Q10) values (Figures 3L,O). It appears that the seed maturation stage is more sensitive to the changing temperatures than traits at heading, specifically heading time and branch number at heading. While seed maturation time or seed weight are strongly affected by temperature, this is not reflected in seed area. This suggests that it is the composition of the seeds that has changed and not the size. For seed area, Mon3 and Bd21 do show a significant difference between high and low temperatures (Figure 3P). However, seed area shows no correlation to temperature among accessions (Figure 3Q) and seed area shows no temperature sensitivity as indicated by the log(Q10) that is zero on average and shows low variability (Figure 3R).

### Cool Temperatures Increase Dormancy of Progeny Seed and Accelerate Germination

**FIGURE 4:**
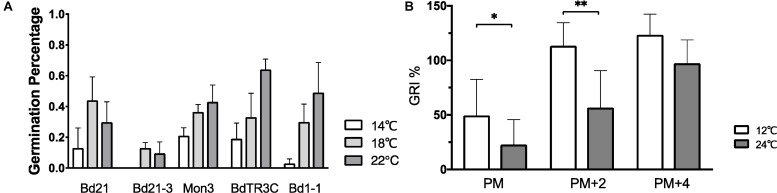
The effect of different temperatures on seed dormancy. **(A)** Germination percentage of seeds (Bd21, Bd21-3, Mon3, BdTR3C, and Bd1-1) produced at 14°C (white bar), 18°C (gray bar), and 22°C (black bar) incubated at 12°C. **(B)** Germination rate index of seeds (physiologically mature seeds (PM), 2-week physiologically mature seeds (PM + 2) and 4-week physiologically mature seeds (PM + 4) from twenty natural accessions were incubated at 12°C (gray bar) and 24°C (black bar). The accessions information mentioned in Supplementary Table S1. One-way ANOVA was used for statistically analysis and ^*^adjusted *P* < 0.05, ^∗∗^adjusted *P* < 0.01.

In order to explore how ambient temperature during seed development affects seed dormancy, physiologically mature seeds from five accessions grown at 14, 18, and 22°C were harvested and incubated at 12°C for seed germination. The percentage of germinated seeds after 10 days was used to measure seed dormancy. No clear conclusion can be made in all accessions, but a similar pattern is observed within one accession, in which elevated temperatures increase the seed germination percentage (Figure 4A). However, no statistically significant difference was observed between different temperatures within one accession. While the plants were grown in well-controlled environmental conditions, the germination percentage between plants varies strongly (large error bar). This is also related to the fact that Brachypodium plants produce only a limited number of seeds in comparison to Arabidopsis. Despite this, one can still see the clear pattern between different temperatures. More replicates should be used in further research because of the large variance.

The average GRI of at least fifteen different accessions was calculated to evaluate the temperature effects on seed germination (Figure 4B and Supplementary Table S1). A low GRI indicates slow germination speed. We compared the GRI at 12 and 24°C to test the germination of physiologically mature, 2-week after ripened or 4 week after ripened physiologically mature seeds, with after ripening occurring at 37°C. We find that imbibition at low ambient temperature accelerates physiologically mature seed germination. A higher incubation temperature slows down germination of physiologically mature or 2-week physiologically mature seeds, while there is no difference of temperature responsive germination on the fully after ripened seeds (Figure 4B).

### Temperature and Phenology

**FIGURE 5:**
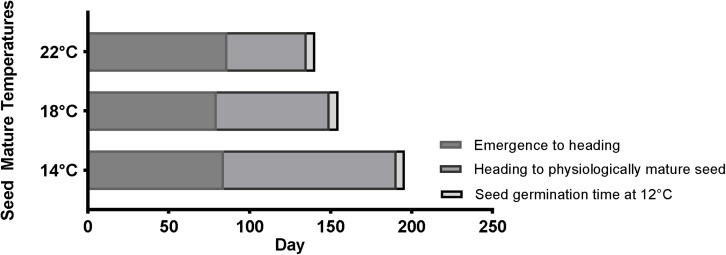
Summary for the timing of average emergence to heading, average heading to physiologically mature seed and average seed germination time (12°C) of five accessions (Bd21, Bd21-3, Mon3, BdTR3C, and Bd1-1) at 14, 18, and 22°C.

In order to understand how temperature affects the life cycle of Brachypodium, the average length of different developmental stages among different accessions, including Bd21, Bd21-3, Mon3, BdTR3C, and Bd1-1, was summarized in Figure 5. The MGT of physiologically mature seeds was calculated as germination time (Supplementary Figure S1). In general, a cool temperature extends the length of the life cycle in Brachypodium, while a warm ambient temperature shortens the life cycle. Figure 5 clearly shows that the length of the life cycle is most strongly dependent on the seed maturation time. However, as we described above, this can vary strongly for individual plants when seed germination is considered because seed dormancy varies widely between seeds produced by the same plant.

## Discussion

Like all organisms, plant life is highly influenced by temperature. Depending on the range, temperature can cause plants to flourish or result in their detriment through frost and heat stress (Kotak et al., 2007; Barnabás et al., 2008; Farooq et al., 2011). In light of impending climate change, the effect of ambient temperatures on plant growth has become a major focus in plant research. In this research, we studied the ambient temperature effects on the different developmental stages in the temperate model Brachypodium. We can conclude that the high ambient temperature (26°C) promotes leaf development in facultative accessions Bd21, Bd21-3, and Mon3, while the leaf development of the winter accession BdTR3C shows no different between 18 and 26°C (Figure 2). This is consistent with the leaf number of BdTR3C at heading, in which no significant difference was observed between different temperatures (Figure 3D). During the reproductive stage, high ambient temperatures promote leaf numbers at heading, shorten the seed set time after heading and decrease seed weight among all accessions. Even though heading time, branch numbers when heading and seed area are not correlated to temperature overall, these traits are still sensitive to temperature in some accessions. Natural variation in temperature sensitivity (Q10) is highly conserved in seed traits, but heading time, branch numbers and seed area show no temperature sensitivity. Bd1-1 contributes to the large variation in Q10 regarding leaf and branch numbers. Seeds produced at low ambient temperatures have higher dormancy than those produced at high ambient temperatures (Figure 4A) and low ambient temperature (12°C) rather than high ambient temperature (24°C) increases the rate of seed germination (Figure 4B). There is however no statistical difference between the seed germination rates of seeds developed at different maternal growth temperatures due to the high levels of variation between replicates. In the whole life cycle of plant growth, seed development appears to be more sensitive to low temperature by extending the seed set period in these conditions, which is consistent with the research published by Boden et al. (2013).

Modeling of early leaf development in cereal crops is used to predict the correct sowing time and to manage crop development in order to achieve maximum crop productivity (Cao and Moss, 1989a; Cao and Moss, 1991). The rate of leaf emergence in cereals is strongly affected by air temperature in the field (Kirby et al., 1985). The developmental rate of leaves in wheat and barley was reported to increase with elevated temperatures before declining beyond an optimum temperature (Cao and Moss, 1989b; Bahuguna et al., 2014). In Brachypodium, leaf development was promoted by high ambient temperature in Bd21-3 (Matos et al., 2014), consistent with what we observe at an early developmental stage (Figure 2B). There is also a growth pattern for early leaf development at high ambient temperature, in which developmental rate is initially accelerated and subsequently decreases before increasing again. To our knowledge, this has not yet been reported in wheat. Based on these results, the leaf development of facultative accessions is more sensitive to ambient temperatures, but the winter accessions may need an optimal temperature range to have a clear growth pattern related to temperature. This effect of temperature on leaf developmental rate is reflected in the correlation between temperature and leaf number at the heading stage. Generally, the high ambient temperature accelerates the leaf number at heading stage.

Flowering late or early can affect crop yield by influencing seed development. For temperate cereals, flowering is adversely affected by the risk of frost, limited precipitation and high temperature during filling time. However, there is no-linear correlation between ambient temperatures and the period from emergence to anthesis (Angus et al., 1981). Thus it seems more important to explore the temperature responses after anthesis (Wardlaw and Moncur, 1995; Ferris et al., 1998; Shi et al., 2016). Similarly, heading time in Brachypodium showed no correlation with elevated temperatures among five accessions. But, there is a significant difference for one accession, Bd21, between temperatures (Figure 3A). Bd21 showed significant differences in heading time between 14 and 18°C, and between 18 and 22°C. In contrast, Bd21 was reported to show no difference in heading time between 22 and 27°C in continuous long days and delayed heading at high temperature rather than low temperature in long day after shifting from short day (Boden et al., 2013). This might imply that there is an optimal threshold temperature which affects the heading time in Brachypodium.

For seed development after anthesis, the temperature effect is highly conserved in cereals and Brachypodium. In summary, warm temperature can shorten seed set time (Sofield et al., 1977; Springthorpe and Penfield, 2015) and largely reduce the seed weight (Al–Khatib and Paulsen, 1984; Wardlaw and Moncur, 1995; Ferris et al., 1998; Huang et al., 2014; Nasehzadeh and Ellis, 2017). This coincides with our results in Brachypodium as well as previous reports (Boden et al., 2013). The temperature effect on seed dormancy is also highly conserved in different species. In wheat, it was reported that higher temperature during seed set time reduces dormancy, and lower temperature during seed germination breaks seed dormancy (Reddy et al., 1985; Nyachiro et al., 2002; Nakamura et al., 2011; Ikić et al., 2012). Though no significant difference was observed in seed dormancy between different temperatures within one accession during seed set period, the pattern that elevated temperature during seed maturation reduces the seed dormancy appears clear (Figure 4A). Large variation between replicates may be one reason for the absence of a statistically significant difference. The germination rate of the freshly harvested Bd21-3 seeds can reach more than 70% when plants were grown at 20°C (Barrero et al., 2011). However, the dormancy of Bd21-3 is quite high in our research (Figure 4A). This may indicate that the important effect of ambient temperature during seed maturation on seed dormancy and the parental environment has the potential to affect germination (Elwell et al., 2011; Springthorpe and Penfield, 2015). Further research may be required to confirm the seed dormancy of Bd21-3 as 14°C inhibits the abnormal development of seeds in this accession. In addition, we found that physiologically mature seeds can germinate better at 12°C compared to 24°C (Figure 4B), which is consistent with results in Arabidopsis (Springthorpe and Penfield, 2015) and wheat (Nyachiro et al., 2002). Thus the temperature effect on seed development and dormancy is similar to other species, in spite of different optimal temperature ranges, and the ambient temperature effect on seed dormancy and germination appears more obvious than that on floral transition.

Arabidopsis has been widely used as a model for more than 30 years (Meinke et al., 1998), which has broadened our understanding of the molecular mechanism of plant development. The molecular mechanisms of ambient temperature responses in Arabidopsis studied so far has revealed several major genes, mentioned previously. However, Arabidopsis cannot represent all plant species and particularly grasses are only distantly related. In temperate cereals, the major regulator might be slightly different. The large genome of wheat and barley slows down the elucidation of molecular mechanisms of ambient temperature responses in cereals. *B. distachyon* has been selected as a model for temperate grasses as early as 2001 (Draper et al., 2001). In this study, we explore the ambient temperature effects on the Brachypodium life cycle and found that temperature responses between cereals and Brachypodium are highly conserved. This shows it to be an appropriate model to explore the mechanism behind the ambient temperature responses in temperate grasses.

## Data Availability

All datasets for this study are included in the manuscript and the Supplementary Files.

## Author Contributions

AK and KG designed and performed the vegetative stage experiments. ML and KG designed and carried out the reproductive stage and seed dormancy experiment. ND and MH contributed to the seed germination data. ML wrote the manuscript. KG and AK revised the manuscript. All authors contributed to the final manuscript.

## Conflict of Interest Statement

The authors declare that the research was conducted in the absence of any commercial or financial relationships that could be construed as a potential conflict of interest.
