# Supplementary material for: The Effect of Ambient Temperature on Brachypodium distachyon Development
Source: Front Plant Sci. 2019 Aug 21;10:1011. doi: 10.3389/fpls.2019.01011 (PMC6712961; doi:10.3389/fpls.2019.01011)
Supplement: Supplementary file 2 [file Table_2.docx]

Supplementary Material

**Supplementary Figure S1.** Mean germination time for physiologically mature seeds produced at 14℃ (white bar), 18℃ (grew bar) and 22℃ (black bar). NA: missing data. One-way ANOVA was used for statistically analysis and adjusted P-value <0.05 marked as ‘*’, adjusted P-value <0.01 marked as ‘**’.
